# Harnessing Mesenchymal Stromal Cells for the Engineering of Human Hematopoietic Niches

**DOI:** 10.3389/fimmu.2021.631279

**Published:** 2021-03-15

**Authors:** Alice Pievani, Roberto Savoldelli, Juliane Poelchen, Elisa Mattioli, Giorgio Anselmi, Alice Girardot, Jochen Utikal, Pierre Bourdely, Marta Serafini, Pierre Guermonprez

**Affiliations:** ^1^Department of Pediatrics, M. Tettamanti Research Center, University of Milano-Bicocca, Monza, Italy; ^2^The Peter Gorer Department of Immunobiology, Centre for Inflammation Biology and Cancer Immunology, School of Immunology & Microbial Sciences, King's College London, London, United Kingdom; ^3^Cancer Research UK King's Health Partner Cancer Centre, King's College London, London, United Kingdom; ^4^Skin Cancer Unit, German Cancer Research Center (DKFZ), Heidelberg, Germany; ^5^Department of Dermatology, Venereology and Allergology, University Medical Center Mannheim, Ruprecht-Karl University of Heidelberg, Mannheim, Germany; ^6^MRC Molecular Hematology Unit, Radcliffe Department of Medicine, Medical Research Council, Weatherall Institute of Molecular Medicine, University of Oxford, Oxford, United Kingdom; ^7^Centre for Inflammation Research, CNRS ERL8252, INSERM1149, Hopital Bichat, Université de Paris, Paris, France

**Keywords:** hematopoiesis, organoids model, bone marrow niche, mesenchymal stroma cell, thymus epithelial cell, 3D culture

## Abstract

Tissue engineering opens multiple opportunities in regenerative medicine, drug testing, and modeling of the hematopoiesis in health and disease. Recapitulating the organization of physiological microenvironments supporting leukocyte development is essential to model faithfully the development of immune cells. Hematopoietic organs are shaped by spatially organized niches defined by multiple cellular contributions. A shared feature of immune niches is the presence of mesenchymal stromal cells endowed with unique roles in organizing niche development, maintenance, and function. Here, we review challenges and opportunities in harnessing stromal cells for the engineering of artificial immune niches and hematopoietic organoids recapitulating leukocyte ontogeny both *in vitro* and *in vivo*.

## Introduction

Mechanistic studies of the human hematopoiesis, drug testing, immunization and regenerative medicine purposes share a need for immune organoids recapitulating physiological immune niches. Hematopoietic organs supporting leukocyte development are shaped by spatially organized areas defined by multiple cellular contributions. All immune niches contain mesenchymal stromal cells (MSCs) endowed with unique and specific roles in organizing niche development, maintenance and function. MSCs were first described in murine bone marrow (BM) as a population of cells containing fibroblast progenitors and capable to differentiate into cartilage, bones, adipocytes, and to recapitulate a hematopoiesis supporting microenvironment upon transplantation (1, 2). MSC features were subsequently identified in non-hematopoietic cells of the human BM niche (2–6). Here the term “MSCs” will be used from a practical perspective, referring to an often heterogenous populations of mesodermal origin, regardless of their multilineage potential and self-renewal capabilities at the single cell level.

In this review we highlight the challenges and opportunities in harnessing MSCs for the engineering of artificial immune niches recapitulating the physiological bone marrow (BM) or thymic niche.

## The Bone Marrow Niche From the MSC Perspective

### MSCs in the Murine Niche

The BM is the primary lymphoid organ deputed to the maintenance, self-renewal and lineage commitment of adult hematopoietic stem cells (HSCs). HSCs originate from the dorsal aorta in the aorta-gonad-mesonephron region of the embryo. Embryonic HSCs seed and expand first in the fetal liver before colonizing specific areas of the trabecular region of the BM called niches where they become proper mature HSCs. Niches are complex 3D environments composed of multiple cell types and factors, such as extracellular matrix (ECM), oxygen tension, soluble molecules, and shear forces, all of which govern the fate of HSCs. Most of our knowledge regarding the components of the BM niche and how they interact to modulate HSC fate has been attained by findings in murine models due to the limited possibilities to access and harness information from human specimens. MSCs are critical contributors of the HSC niche. A key feature of MSCs is the delivery of chemokines and hematopoietic growth factors essential for the maintenance and differentiation of HSCs (2). The chemokine CXCL12 engages the CXCR4 chemokine receptor on HSCs. As a consequence, the CXCR4/CXCL12 axis is essential for HSC retention within the BM niche and maintaining HSC quiescence (7). SCF/KITL engages the KIT tyrosine kinase receptor on HSC which is crucial for their maintenance. Proteolytic cleavage and alternative splicing mechanisms generate membrane bound and secreted forms of SCF/KITL. Selective inactivation of the membrane bound form of KITL in *Sl/Sl*^d^ mutant mice impairs long term HSCs despite the presence of soluble KITL (8, 9). In addition, adult deletion of SCF/KITL in inducible knock-out mice depletes HSCs (10). This highlights the importance of cell-to-cell contacts in the delivery of hematopoietic growth factors to HSCs for their maintenance. The production of niche factors has been an important feature guiding the identification of MSC subpopulations relevant for the HSCs niche. For instance, CXCL12^gfp^ mice allowed to identify CXCL12 abundant fibroblastic reticular cells (CAR) (7), while SCF^gfp^ reporter mice showed SCF/KITL expression in both MSCs and endothelial cells (ECs) (10). In addition to ECs and MSCs, multiple other lineages have been reported to also contribute to the maintenance of the niche: for instance, osteolineage cells, adipocytes, neurons and hematopoietic cells. The endosteal niche is found in proximity of the endocortical region of the internal bone shell. It is formed mainly by MSCs together with osteoclasts, osteoblasts, and tissue resident macrophages (11, 12). Multiple lines of evidence locate the HSCs niches mainly in the perivascular areas of BM. The perivascular niche is formed by endothelial and other MSCs. SCF^gfp^ reporter mice have enabled a systematic evaluation of SCF/KITL-expressing cells and revealed a crucial contribution of endothelial and perivascular MSCs compartments (10).

The precise location of HSCs with respect to sinusoidal ECs (sECs) or arterial ECs (aECs) has been controversial (13–18). It is unclear if sinusoids represent a “proliferative” niche whereas arteriole would represent a “quiescent” niche (15) or if sinusoids are the main HSCs localization regardless of their proliferation status (16). The late conclusion has been provided by examining thoroughly HSCs localization defined by KIT^+^ GFP-labeled cells in alpha-catulin^gfp^ reporter mice (16). The development of a fluorescent reporter mouse line fully specific for long term Flt3^−^ HSCs (MFG mice) has enabled to address their localization in un-manipulated environments by intravital imaging (18). This breakthrough revealed that quiescent HSCs seat mostly in vicinity to sECs (and the endosteum), in mildly hypoxic environment (18). Despite the preferential localization of HSCs next to sECs, sECs have recently been reported to express lower levels of SCF/KITL and CXCL12 as compared to aECs, thereby questioning the involvement of aECs in the niche (19–22). Identifying the MSC population of the HSC niche and defining its developmental potential within mesenchymal lineages has proven to be a major challenge. Early work has evidenced BM mesenchymal osteoprogenitors (i) can be engrafted and regenerate bones (2, 23, 24); (ii) are endowed with a multi-lineage potential for osteoblasts, chondrocytes, adipocytes, and fibroblasts (2, 4). Later studies have attempted to prospectively identify self-renewing and multi-potent *bona fide* MSCs (2). A breakthrough in the field has been afforded by studies which reported that murine CD45-Tie2-${\text{a}}_{\text{v}}^{+}$CD105^+^Thy1.1-fetal BM MSCs are able to recapitulate a functional HSCs niche upon transfer within the renal capsule (25). In addition, Morikawa et al. have shown that PDGFRa^+^Sca1^+^CD45^−^TER119^−^ MSCs from mouse BM give rise to osteoblasts, reticular cells and adipocytes after *in vivo* transplantation (26, 27). Taken together, these studies have defined the so-called “two stem cells” paradigm in which multipotent MSCs are both organizers and components, besides HSCs, of the BM niche (25, 28, 29). The Frenette lab has shown that Nestin^gfp^ reporter mice could be instrumental to identify MSCs with stem cell properties: (i) high CFU-F activity; (ii) a multi-lineage potential to generate fibroblasts, osteoblasts, chondrocytes and adipocytes (in both mice and humanized mice); (iii) a self-renewal potential upon transfer (28). More recently, MSCs activity has been tracked using the LepR^cre^ mice targeting MSCs expressing the leptin receptor (30, 31). Recent scRNAseq studies have re-evaluated the heterogeneity among LepR^+^ cells and highlighted the existence of two subsets, one expressing adipocyte-related genes (Adipo-CAR) while the other expressing osteogenic genes (Osteo-CAR) (19, 21). Micro-dissection analysis suggests that Adipo-CAR would be more associated to sinusoids, as compared to Osteo-CAR, preferring arteriole and other regions (21, 22). Multiple line of evidences, including the inference of developmental trajectories *in silico* (32), suggest that the Adipo-CAR fraction of LepR^+^ cells contains the most primitive MSC progenitors activity giving rise to multiple adipocytes, osteoblasts and chondrocytes (22, 32). Studies from the Morrison lab have established that LepR^+^ cells account for most BM CFU-F activity, represent the main source of bone and adipocytes in adult BM and can give rise to cartilage (30). Various technical approaches have identified multiple cellular entities within the MSCs compartment proposed to play a major role in shaping of the perivascular niche (10, 15, 28, 33):

- Arteriole-associated pericytes. Pioneer work from the Frenette lab identified arterioles-associated pericytes expressing NG2/CSPG4 coupled with quiescent HSCs in the endosteum region (15). NG2^+^ pericytes are rare and display bright GFP levels in *Nestin*^gfp^ reporter mice (*Nestin*^bright^) (15). Conditional and inducible cellular deletion of NG2 pericytes (using NG2-*cre*^ERTM^xROSA^iDTR^ mice) induces HSCs cycling and reduces long-term repopulating activity (15). *Nestin*^bright^ pericytes express high levels of CXCL12. This feature is relevant for HSCs niche, as conditional inactivation of CXCL12 -but not SCF- in NG2^+^ pericytes reduces HSCs numbers, perturbs their localization within the BM and induces their peripheral mobilization (34).
- Sinusoid-associated pericytes. Sinusoid associated MSCs display high level of GFP in *Cxcl12*^gfp^ mice (7), intermediate level of GFP in *Nestin*^gfp^ reporter mice (15), and are labeled in *LepR*^cre^ mice (10, 30). From the niche point of view, LepR^+^ positive cells (labeled in *LepR*^cre^ mice) express high levels of SCF/KITL and some levels of CXCL12 (10, 33). Conditional deletion of SCF in LepR^+^ cells depletes quiescent HSCs (10). Conditional inactivation of CXCL12 in LepR^+^ cells triggers HSCs mobilization in periphery (33). In addition to SCF and CXCL12, Adipo-CAR produce multiple hematopoietic factors like IL-15, IL-34, Csf1, Bmp4, Ccl19, and Ccl2 (22). Furthermore, LepR^+^ cells act also as a major source of IL-7, important for the homeostasis of lymphoid-committed progenitors which also express CXCR4, ensuring their correct positioning by responding to CXCL12 from LepR^+^ MSCs (35).

### MSCs in the Human Niche

Compared with the mouse system, much less knowledge exists regarding the architecture of the human BM niche and the function of its different cellular components. Using human bone biopsy specimens, Guezguez et al. provided evidence of HSC propensity to localize to endosteal regions of the trabecular bone area (36). CD34^+^ cells follow a spatial gradient within the marrow cavities with a maximal concentration in the first 50 μm from the bone trabecula surfaces where the blood vessels are most concentrated (37). Efforts in recent years have also been made to identify the specific human MSC subpopulation that supports HSC activity. In the human BM, a multi-potent CD45^−^CD105^+^CD146^+^ sinusoid-associated fraction of MSCs was shown to be able to recapitulate a functional HSCs niche upon transfer under the mouse skin (5). Human pericytes CD146^+^ express nestin, CXCL12, and LepR similar to mouse perivascular MSCs and directly support the *ex vivo* maintenance of human HPSCs through cell-to-cell contact and activation of Notch signaling (38). A fraction of human CD146^+^ perivascular MSCs expressing PDGFRα, CD51, and multiple niche factors (e.g., CXCL12, SCF, and angiopoietin1) would correspond to an ortholog of GFP-positive cells in Nestin^gfp^ mice. Upon transplant in immunodeficient mice, this CD146^+^ MSC subset is mainly localized in close proximity to mouse sinusoids and recruit hematopoietic cells (39). All these evidences seem to suggest that CD146^+^ perivascular cells could represent the human counterpart of the CAR cells or nestin^+^ cells described in the mouse. More recently, a population of CD146^−^ CD271^+^ MSCs localized in the trabecular region of the human BM has been identified. Like the CD146^+^ perivascular cells, these CD271^+^ MSCs showed high clonogenicity, trilineage differentiation capacity *in vitro*, and ability to transfer a HSC microenvironment upon transplantation (40). CD271^+^SSEA4^+^ MSCs have also been shown to express high levels of HSC-supportive genes and to support HSC engraftment potential (41). These data indicate that different subtypes of MSCs exist in the human BM niche and interact with HSCs in specific regions.

## Modeling the Bone Marrow Niche Using MSCs

### A Role for MSCs in Modeling the Niche *in vitro*

HSCs *in vitro* generation is instrumental to understand hematopoiesis as well as to model genetical disorders and cancers. Moreover, large scale manufacturing of HSCs could represent a valuable therapeutic option for many patients. To date, two different approaches have been attempted in this way: (i) the expansion of large number of HSCs from BM or umbilical cord blood (CB) (42, 43); (ii) the *de novo* generation of HSCs from induced pluripotent stem cells/embryonic stem cells or via somatic cell reprogramming (44–48).

Standard culture protocols supporting the proliferation of long term *bona fide* engraftable, self-renewing hematopoietic stem and progenitor cells (HSPCs) with multi-lineage potential remain a challenge. Given the natural role of MSCs in the HSC niche, co-culture with MSCs is a very popular approach to maintain and expand HSCs *in vitro*. However, these conventional systems fail at reproducing the complexity of the BM niche.

The first step in mimicking the physiological HSC niche consist on generating a 3D environment using different biomaterials, such as hydrogels, silicate structures, and human bone-derived scaffolds (49) (Table 1).

**Table 1:** Modeling the BM niche *in vitro* using human MSCs.

**Reference**	**Scaffold-based culture system**	**Hematopoietic cell source**	**Findings**
Feng et al. (50)	Fibronectin (FN) or collagen-conjugated 3D polyethylene terephthalate (PET) scaffold	CB-derived CD34^+^ HSPCs	Significant expansion of CD34^+^ cells with high SCID repopulating function
Nichols et al. (51)	Silicate scaffold coated with PDDA and clay and seeded with stromal cells (human bone marrow stromal HS-5 cell line and human fetal osteoblast 1.19 cell line)	CD34^+^ HSPCs from different sources	Support expansion of HSPCs and production of functional B cells
Rödling et al. (52)	3D macroporous PEG hydrogel with RGD-peptides seeded with human MSCs and perfused in a bioreactor	CB-derived CD34^+^ HSPC	Maintenance and differentiation of CD34^+^ HSPCs in dynamic culture. Importance of perfusion on drug testing (myelotoxic effects of chemotherapeutics)
Braham et al. (53)	Bio printable pasty CPC scaffold with seeded O-MSCs to model the endosteal niche, and Matrigel containing both EPCs and MSCs to model the perivascular niche	Primary CD138^+^ myeloma cells	Significant increase in the proliferation of myeloma cells. Essential role of the perivascular niche over the endosteal niche in supporting myeloma cells
Ferreira et al. (54)	Comparison of 3D PCL, PLGA, fibrin and collagen scaffold either seeded or not with UC-MSCs	CB-derived CD34^+^ HSPCs	All scaffolds except PLGA favored the expansion of HSPCs. When the scaffolds are seeded with MSCs the results improve, electing fibrin as the best scaffold
Raic et al. (55)	3D macroporous hydrogel scaffold seeded with UC-, BM-MSCs, or osteoblast-like cells	CB-derived CD34^+^ HSPCs	HSPCs cultured with BM-MSCs in 3D systems have the highest proliferative status while maintaining stemness
Leisten et al. (56)	3D collagen scaffolds in suspension to generate a double niche, in semi-solid and liquid phase	CB derived HSPCs	Most differentiated cells are found in the liquid phase niche. Differentiation is boosted by UC-MSCs. More immature HSPCs relies in the solid phase of the scaffold
Bourgine et al. (57)	Porous hydroxyapatise scaffold seeded with BM-MSCs within a perfusion bioreactor	CB-derived CD34^+^ HSPCs	Supported maintenance of HSPCs; possibility to perturb HSPCs behavior by molecular customization or injury stimulation
Sieber et al. (58)	Hydroxyapatite coated zirconium oxide scaffold seeded with BM-MSCs in a microfluidic system	CB-derived CD34^+^ HSPCs	Successful long-term culture (up to 28 days) of HSPCs with multilineage differentiation potential
Bruce et al. (59)	3D microfluidic model loaded with BM-MSCs and osteoblasts encapsulated in collagen matrix	B-ALL SUP-B15 cell line	Decreased chemotherapeutic drug sensitivity of leukemic cells in 3D tri-culture model from the 2D models
Chou et al. (60)	Perfused PDMS organ chip with “hematopoietic” channel (filled with BM-MSCs in a fibrin gel) and “vascular” channel (lined by HUVECs)	mPB-derived CD34^+^ HSPCs Shwachman-Diamond Syndrome BM-derived CD34^+^ HSPCs	Supported differentiation of multiple blood-cell lineages; reproduction of hematotoxicities after chemotherapy/ionizing irradiation; reproduction of marrow recovery after drug-induced myelosuppression; recapitulation of hematopoietic abnormalities of patients with genetic disorders

*HSPCs, hematopoietic progenitor stem cells; HUVEC, Human Umbilical Vein Endothelial Cells; PDDA, poly(diallyldimethylammonium chloride); CPC, calcium phosphate cement; O-MSCs, osteogenic multipotent mesenchymal stromal cells; BM, bone marrow; MSC, mesenchymal stromal cells; PDMS, poly(dimethylsiloxane); PEG, polyethylene glycol; RGD, arginylglycylaspartic acid; EPC, endothelial progenitor cells; PCL, poly(epsilon-caprolactone); PLGA, poly (lactide-co-glycolide) acid; UC, umbilical cord; CB, cord blood; PB, peripheral blood; B-ALL, B-cell acute lymphoblastic leukemia*.

Those polymers provide structure and support for cell proliferation but also a spatial control of the cell interactions. This is achieved by physical limitation in cell-to-cell contact and controlled availability of soluble factors. Integration of ECM within these scaffolds has been explored. Feng et al. demonstrated how a polyethylene terephthalate (PET) scaffold can be engineered with ECM proteins, such as collagen and fibronectin, to support the expansion and differentiation of CB-CD34^+^ cells (50). Importantly, those synthetic scaffolds can be colonized with different types of MSCs together with ECs to mimic *in vivo* niches. Ferreira et al. and Raic et al. (54, 55) have developed two independents models based on porous 3D scaffold for the expansion of HSCs *in vitro*. Ferreira et al. tested several natural polymers as scaffolds in combination with MSCs as support and found that 3D fibrin scaffold seeded with MSCs is the most efficient system to expand CD34^+^ cells. Importantly, expanded HSPCs maintain a more primitive immunophenotype and exhibit strong engraftment and multi-organ repopulation capability (54). Similarly, Raic et al. showed that the positive effect of MSCs on preservation of HSPCs stemness was more pronounced in a porous 3D hydrogel scaffold in comparison to standard 2D culture systems (55). Moreover, phenotypically immature HSPCs (CD34^+^CD38^−^) with self-renewal and repopulation capacity are shown to be maintained in 3D collagen co-culture with MSCs, in close proximity to the collagen fibers (56). Nichols et al. demonstrated how a silicate and clay 3D structure seeded with heterogeneous stromal cells and human HSCs is able to expand the HSCs and promote B cell development after 28 days, with a significant increase compared to its 2D counterpart (51).

Latest development in the field is the combination of 3D organoids with perfusion-based bioreactor systems, the so-called “4D system,” to further increase the amount of resemblance to *in vivo* niches. The BM microenvironment is indeed strictly regulated by the concentration of soluble factors, oxygen levels, and the mechanical stress applied by blood flow. The use of bioreactors and microfluidics devices can than allow modeling the niche situation more closely. Rödling et al. developed a bioreactor system for perfusion of 3D scaffolds seeded with MSCs mimicking the BM *in vivo* and demonstrated the importance of perfusion during drug treatment as results are different with and without perfusion (52). Indeed, while under static conditions the more mature CD34^−^ subpopulation was more sensitive to 5-fluorouracil treatment, under dynamic conditions both CD34^−^ and CD34^+^ cells responded similarly. Bourgine et al. reported the engineering of BM-like tissues in a perfusion bioreactor system partially recapitulating structural, compositional and organizational features of the native human osteoblastic niche environment, resulting in the support of HSPC functions. Their approach consists in the use of bone-like porous hydroxyapatite scaffold functionalized by MSCs and osteoblastic cells and by the ECM they deposited during perfusion culture in bioreactors (57). 3D scaffold-based microfluidic chips have been introduced for the generation of a “BM-on-a-chip.” Torisawa et al. in a pioneer murine study showed that PDMS (poly-dimethyl-siloxan) device loaded with bone forming factors could be seeded *in vivo* by BM-MSCs, such as CXCL12^+^ CAR and Nestin^+^ LepR^+^ perivascular MSCs. *Ex vivo* culture of the organoid can be achieved in a microfluidics chip (61). Sieber et al. loaded hydroxyapatite-coated zirconium oxide scaffold with human MSCs in a microfluidics chip. This enables HSPCs to maintain multilineage potential up to 28 days *in vitro* (58). Chou et al. seeded a fibrin scaffold with MSCs and CD34^+^ cells in a microfluidics device embedding vascular channel seeded with human ECs. Upon 4 weeks of culture the vascularized chip recapitulated the ontogeny of multiple blood cell lineages while maintaining undifferentiated HSPCs. Moreover, this system has been used to model the impact of a genetic disorder on myelopoiesis (60).

MSCs in microfluidic devices have also been instrumental in modeling the leukemic niche *in vitro* for the sake of studying chemotherapeutics and immunotherapy (53, 59, 62).

### Engineering MSCs to Regulate HSC Self-Renewal or Direct Hematopoietic Differentiation

**Figure 1:**
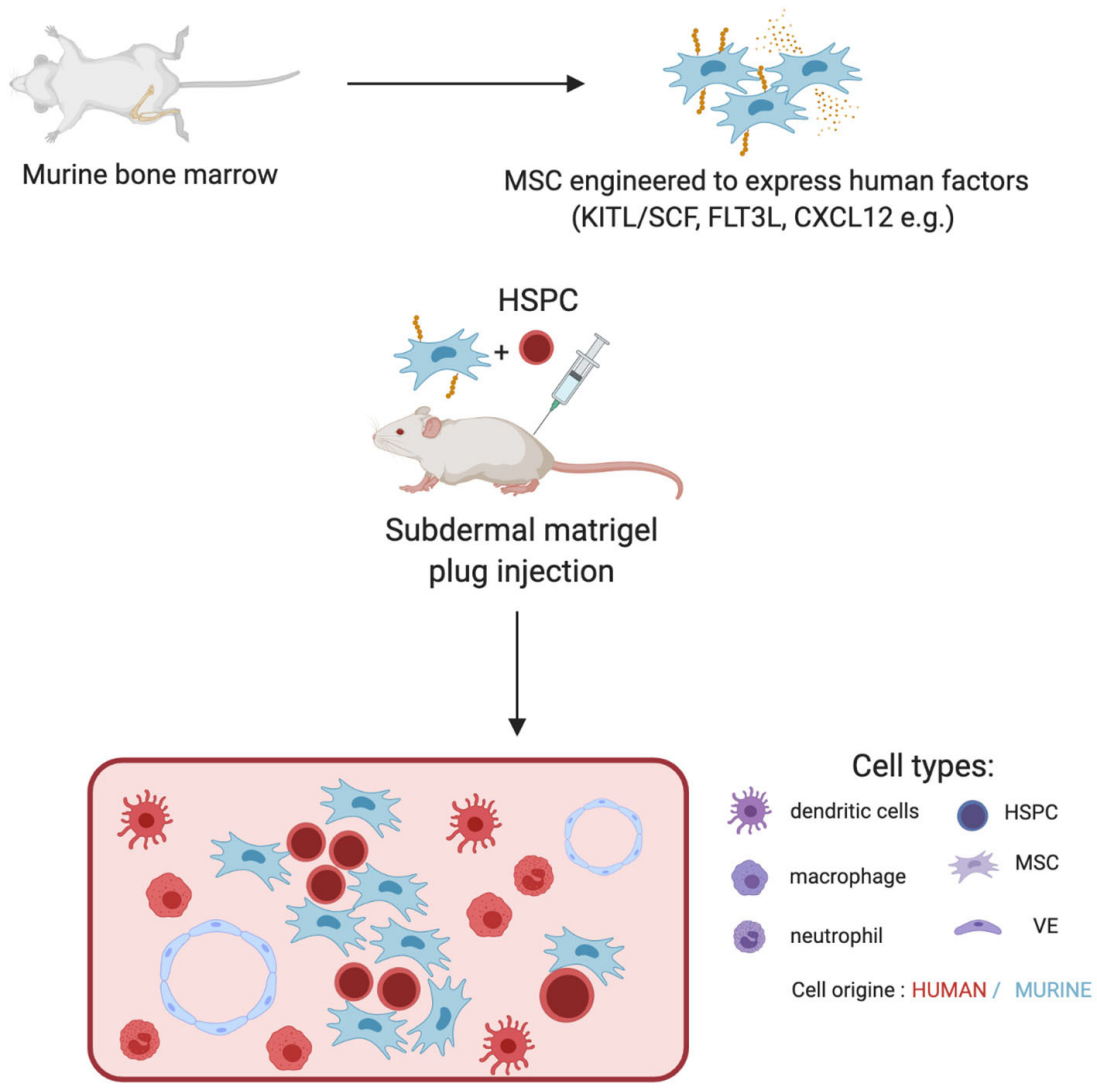
Engineering of murine MSCs to recapitulate human hematopoiesis. MSC from murine bone marrow are isolated and expanded *ex-vivo*. Those are subsequentially engineered to express human factors and injected together with human HSPCs into the back of an NSG mice in Matrigel plugs. The plug is retreated 2 weeks later. It displays an unorganized structure containing murine MSCs and vasculature together with human hematopoietic progeny. Progeny is dependent on the factors expressed by MSCs.

By virtue of their role in HSC niche, engineering of MSCs offers a window of opportunity to control HSC self-renewal or drive hematopoietic differentiation. Several factors regulate the HSC-supporting activity of MSCs. For instance, primary Nestin^+^ murine MSCs rapidly lose their hematopoietic supporting potential upon *ex vivo* culture (63). The transcriptional down-regulation of key niche factors (SCF, ANGPT1, CXCL12, VCAM1) is underlying this process (63). An elegant genetic screen has revealed that overexpression of defined transcription factors (Klf7, Ostf1, Xbp1, Irf3, Irf7) can “revitalize” the niche promoting activity of *ex vivo* cultured primary MSCs. MSCs overexpressing those transcription factors become able to maintain transplantable HSCs (63). This important study opens new avenues in the engineering of MSCs. Some niche factors involved in the hematopoietic hierarchy present a limited sequence homology between mouse and human genes. This is limiting the reactivity across species thereby rendering mouse MSCs sub-optimal for applications involving human HSPCs (64, 65). For these reasons, multiple groups have attempted the expression of defined human hematopoietic factors in murine MSCs lines (Table 2). For instance, co-expression of IL-7 and FLT3L synergizes with DLL1 expression in OP9 to induce the proliferation of T cell progenitors (66). Building on the MS5 mouse MSC line, Anselmi et al. have developed a screening for combination of human niche factors promoting the efficient generation of dendritic cells (DCs) from human CB-CD34^+^ cells (68). They have found that a combination of membrane-bound FLT3L and SCF and soluble CXCL12 is efficient in promoting the differentiation of DCs resembling their circulating counterparts (68). Importantly, transwell experiments indicate that this system relies on the establishment of cell-to-cell contacts. Subcutaneous engraftment of engineered MSCs in basement membrane matrix (Matrigel®) in NSG mice defines a niche supporting both the maintenance of a pool of undifferentiated CD34^+^ cells and the differentiation of DCs (68). Within this niche, it was found that poorly differentiated human CD34^+^ cells would develop cell-to-cell contact with engineered MSCs. Previous reports have established that membrane-bound forms of hematopoietic growth factors like SCF would be specifically required for niche function *in vivo* (8, 9, 69–71). A practical consequence of this is that engineering of MSCs for the over-expression of membrane-bound SCF (or FLT3L) is an attractive strategy to improve the niche-promoting activity of MSCs *in vitro* or *in vivo* (68, 72) (Figure 1). In the same vein, human BM-MSCs have been engineered by Carretta et al. to over-express IL-3 and thrombopoietin (TPO). IL-3/TPO over-expressing MSCs displayed an increased ability to drive the *in vitro* expansion of CD34^+^ cells and improved capacity to support *in vivo* growth of CD34^+^ progenitors expressing the MLL-AF9 fusion gene in a humanized scaffold xenograft model (67). *In vivo* delivery of engineering stromal cells could be improved by the implementation of chemically defined scaffold. For instance, Tavakol et al. have shown that collagen coated carboxyl methyl cellulose micro scaffold (CCMs) seeded with OP9 and HSPCs *in vitro* supports the long term maintenance, over 12 weeks, upon engraftment in immunodeficient mice (73).

**Table 2:** Engineering of MSCs for enhanced human niche activity.

**Reference**	**Cell culture**	**Ectopic expression on stromal cells**	**Findings**
Patel et al. (66)	BM-derived CD34^+^ HSPCs co-cultured with OP9-DL1 cell line	IL-7 and FLT3L	T cell progenitor proliferation
Carretta et al. (67)	CD34^+^ HSPCs co-cultured with human MSCs	IL-3 and TPO over-expression	*In vitro* expansion of CD34^+^ HSPCs. *In vivo*, humanized models producing IL3/TPO support growth of patient samples
Anselmi et al. (68)	CB-derived CD34^+^ HSPCs co-cultured with MS5 or OP9 cell lines	FLT3L, SCF and CXCL12	*In vitro* formation of DCs resembling their circulating counterparts. *In vivo* formation of a niche supporting the differentiation of DCs and the maintenance of undifferentiated HSPCs

*BM, bone marrow; HSPCs, hematopoietic progenitor stem cells; MSC, mesenchymal stromal cell; CB cord blood; IL-7, Interleukin-7; FLT3L, FMS-like tyrosine kinase 3 ligand; IL-3, Interleukin-3; TPO, thrombopoietin; SCF, stem cells factor; CXCL12, C-X-C motif chemokine ligand 12; DCs, dendritic cells*.

Altogether those approaches highlight the versatility of MSC engineering to control and direct HSPCs fate in health and diseases.

### A Role of MSCs in Recapitulating the Human Bone Marrow Niche *in vivo*: The Humanized Ossicle Models

Pioneer studies showing that human MSCs can establish a hematopoietic microenvironment upon transplantation in rodents at non-skeletal sites date back nearly 50 years (74–76). The evolution of bone tissue engineering strategies together with the identification of the human specific osteoprogenitor subpopulations associated with the formation of ectopic bone and bone marrow have been fundamental steps for the generation of humanized BM tissues in mice, the so-called “ossicle” model (5, 25, 28, 29). These human MSC–generated organoids are tissue-specific chimeras, as bone, myelo-supportive stroma, and adipocytes derived from donor MSCs, while blood vessels and hematopoietic tissues are derivatives of host tissues, and they are harmoniously integrated into an overall tissue structure. Several versions of heterotopic transplantation assays have been used, which differ from one another concerning the site of grafting, such as kidney, subcapsular space (25, 77), intramuscular (78), and subcutaneous tissue (79) or type of osteoconductive scaffold/material employed as a carrier (79).

**Figure 2:**
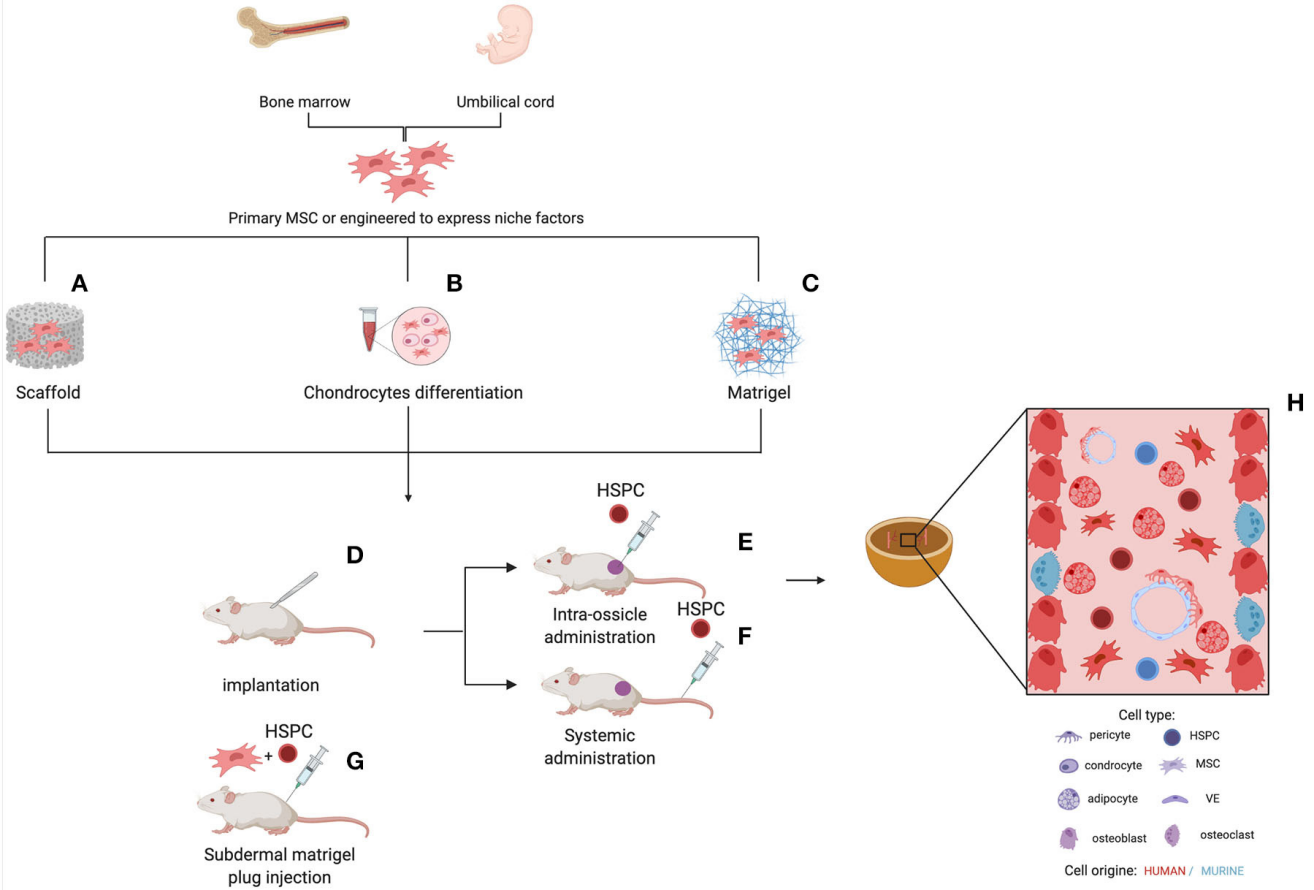
Different strategies for the generation of humanized ossicles. *In vitro* expanded human MSCs (from healthy donors or patients) are seeded onto a scaffold, **(A)** primed to differentiate in cartilage **(B)** or cultured on Matrigel® **(C)** Some protocols include the co-seeding of human ECs and the supplement of osteogenic factors, such as BMPs and PTH. Following the first *in vitro* step, the implantable structures are transplanted subcutaneously into the back of immunodeficient mice for the ossicle formation **(D)**. Aberrant/normal human HSPCs can be added to the system by direct intra-ossicle transplantation **(E)** or intravenous injection **(F)** either before or after the implantation of the ossicle. Irradiation is usually performed to promote engraftment. As an alternative, HSPCs can be seeded onto the Matrigel® plug before the *in vivo* implant **(G)**. The whole process can take several months. The resulting ossicle collected from the mouse is composed of both myelo-supportive marrow stroma and hematopoietic tissues and its progeny of human origin **(H)**. Sinusoidal endothelium, nerve fibers, residual hematopoietic cells, and osteoclasts are derivatives of mouse host.

The next level of humanization of human MSC–generated ossicles has been the introduction of human HSCs. In 2010, Vaiselbuh et al. for the first time reported the successful engraftment of human hematopoietic cells in an ectopic humanized niche obtained implanting subcutaneously in immunocompromised mice polyurethane scaffolds coated with human MSCs, giving rise to the first fully humanized bone/BM organoid model (80). The establishment of a functional human BM niche that could support the maintenance of human blood cells opened the possibility to bridge gaps between the interspecies divergence at a molecular and cellular level in the hematopoietic niche and aspires to become an advanced model to investigate human hematopoiesis and leukemogenesis. Although many aspects of the hematopoiesis are conserved between mice and humans, several differences need to be taken into consideration before applying results obtained in the mouse to humans, specifically for studies aimed at preclinical testing of new therapies (81). Several humanized ossicle models have been reported to date and each has its specificity, as extensively reviewed recently (Figure 2) (82, 83). Current strategies differ for human stromal cell types, carrier material scaffold, human hematopoietic transplantation protocols, and experimental time frames (Table 3). Most protocols use stromal cells derived from the BM of healthy donors. Reinisch et al. suggest that only MSCs, as opposed to the umbilical cord-, skin-, or white adipose tissue-derived MSCs, possess the capabilities to form ectopic bone and BM *in vivo* (77). However, other studies reported the formation of ectopic BM niches through endochondral ossification using stromal cells from different sources, such as cord blood (94) and adipose tissue (97), when primed toward chondrogenesis in the presence of transforming growth factor-β *in vitro* before implant. In most protocols, cells are seeded onto ceramic, collagen, calcium phosphate, or hydroxyapatite-based scaffolds or hydrogels before implantation. These scaffolds provide instructive cues to ensure osteogenesis and represent a 3D template that supports the formation of a bone organ. According to their composition and degradation properties, scaffolds can be entirely remodeled during the ossicle formation or remain part of the organoid structure. The persistence within heterotopic ossicles of artificial, mineralized scaffold material that are not resorbable, is not desirable. It prevents the establishment of the completely normal architecture of bone marrow and complicates the analysis of stromal and hematopoietic cell populations contained within the ossicle, particularly their quantitative assessments. The transplantation of cartilage pellets made *ex vivo* by MSCs consent to avoid these limitations due to the use of exogenous scaffold (91, 93). Stimulating factors, such as BMP2 (96), BMP7 (95), or parathyroid hormone (PTH) (87) can be used to promote osteoblast differentiation of MSCs for the successful *in vivo* formation of mature bone and BM tissues. Furthermore, MSCs have also been genetically modified to express BMP2 (98) or BMP7 (99) generating new bone *in vivo*.

**Table 3:** Humanized ossicle models.

**References**	**Scaffolds**	**Cell source**	**Implant generation**	**Mice strain**	**Time after implant before human blood cells transplantation**	**Conditioning**	**Route of hematopoietic transplantation**	**Transplanted human blood cells**	**Engraftment period**
Vaiselbuh et al. (80)	Polyurethane discs	BM-MSCs (10 × 10^∧^6)	Seeding on scaffold and culturing in medium + 20% FBS + SDF-1 for 4–5 days	NOD/SCID	Unknown	None	*In situ* injection	Primary AML samples	1, 4, 8, 16, 20 weeks
Lee et al. (84)	Polyacrylamide hydrogel	BM-MSCs (1–5 × 10^∧^5)	Seeding on scaffold and culturingin medium + 10% FCS for 1–3 days	NSG or Nu/Nu	4 weeks	Sublethal irradiation	Intravenous injection	CD34^+^ HSPC	16 weeks
Chen et al. (85), Jacamo et al., (86), and Reinisch et al. (87)	Matrigel	BM-MSCs (1.5 × 10^∧^6) and ECFCs (1.5 × 10^∧^6); BM-MSCs (2 × 10^∧^6)	BM-MSCs are mixed with ECFCs in Matrigel immediately before implant; daily PTH administration for 28 days in ossicle-bearing mice	NSG	8–10 weeks	Sublethal irradiation	*In situ* injection; intravenous injection	CB derived-CD34^+^ HSPC or MOLM13 leukemia cells; NALM6 leukemia cells; primary AML, APL, and MF samples	4–11 weeks for CD34^+^, 2 weeks for MOLM13; 10 days for NALM6, 7–24 weeks for patient samples
Groen et al. (88), Antonelli et al. (89), Sontakke et al. (90), and Carretta et al. (67)	BCP	BM-MSCs; IL-3- and TPO-expressing BM-MSCs	Seeding on scaffold and culturing in osteogenic induction medium for 7 days	RAG or NSG	6–8 weeks	None	Intracardiac or *in situ* injection; intravenous injection	CB derived-CD34^+^ HSPC or primary multiple myeloma cells; CB-CD34^+^ BCR-ABL or MLL-AF9 transduced; primary AML or CML samples	8 weeks; from 14 to 38 weeks for AML
Scotti et al. (91), Fritsch et al. (92), and Bourgine et al. (6)	Collagen sponges	BM-MSCs (2 × 10^∧^6)	Seeding on scaffold and culturing for 3 weeks in chondrogenic medium, followed by another 2 weeks of culture in hypertophyc medium	STRG or MISTRG	4 or 6 weeks	Sublethal irradiation	Intravenous injection	CB derived-CD34^+^ HSPC	8 or 6 weeks
Serafini et al. (93) and Pievani et al. (94)	None	BM-MSCs or CB-BFs (3 × 10^∧^5)	Culturing for 3 weeks in chondrogenic medium supplemented with TGF-B1 as pellet	SCID/beige	3 weeks	Sublethal irradiation	Intravenous injection	CB derived-CD34^+^ HSPC	6 weeks
Holzapfel et al. (95)	Tubular mPCL	BM-MSCs (3 × 10^∧^5)	Seeding on scaffold and culturing in medium + 10% FCS for 4 weeks, followed by 4 weeks of dynamic cell culture in osteogenic medium	NSG	10 weeks	Sublethal irradiation	Intravenous injection	Pelvic BM derived-CD34^+^HSPC and CD34^−^ cells	5 weeks
Abarrategi et al. (96)	Collagen sponges	BM-MSCs (1 × 10^∧^5)	Seeding on scaffold and culturing in medium + 10% FBS supplemented with rhBMP2 for 3–7 days	NSG	48 h or 6–4 weeks pre-implant	None or sublethal irradiation	Pre-seeding in the scaffold or *in situ* injection or intravenous injection	CB derived-CD34^+^ HSPC or patient AML samples	12 weeks

*BCP, biphasic calcium phosphate; mPCL, medical grade polycaprolactone; BMSCs, bone marrow mesenchymal stromal cells; ECFCs, endhotelial colony-forming cells; TPO, thrombopoietin; CB-BFs, cord blood borne-fibroblasts; FBS, fetal bovine serum; SDF-1, stromal derived factor 1; FCS, fetal calf serum; PTH, parathormone; TGF-β1, transforming growth factor β1; NSG, NOD/SCID/IL-2rγ^null^ mice; STRG, Rag2^−/−^IL2rγ^−/−^ mice expressing human TPO and SIRPα; MISTRG, Rag2^−/−^IL2rγ^−/−^ mice expressing human TPO, M-CSF, and SIRPα; CB, cord blood; AML, acute myeloid leukemia; APL, acute promyelocytic leukemia; ML, myelofibrosis; CML chronic myelogenous leukemia; HSPC, hematopoietic progenitor stem cells; BMP2, bone morphogenetic protein 2*.

Human blood cells (healthy or malignant) can be administered either by peripheral (tail vein or retro-orbital) or intra-ossicle infusion for organoids of larger dimensions. By contrast, Abarrategi et al. proposed co-implantation of MSCs and human CD34^+^ cells within a collagen sponge, thus avoiding the requirement for subsequent transplantation (96). Conditioning before transplantation does not seem to be a requirement for the successful engraftment of ossicles within immunodeficient mice. The human hematopoietic cell transplantation was generally performed within 3–10 weeks after the *in vivo* implant of the ossicles. Four weeks represent the minimum period required for the formation of marrow cavities allowing human engraftment, through osteoclasts resorption of mineralized tissue within the ossicles (93). Engraftment assessment post-transplantation was achieved from 4 to 38 weeks, depending on the type of cells transplanted (malignant cells with fast/slow engraftment) and the functional readout targeted (short/long-term HSCs engraftment). However, numerous challenges remain to be solved and the variability of different protocols does not allow to do a comparison between different systems in terms of accurate reconstitution of the human niche and potential for human blood engraftment sustaining. The topic has been recently well-reviewed by Dupard et al. (82). Despite the optimization efforts, the ossicles remain largely chimeric as both the nervous system and blood vessels are of mouse origin, although human mesenchymal perivascular cells were detected (6). This implies that investigations on the role of specific niche cellular factors or cytokines should consider the influence of their murine counterparts. Human vascular structure can be generated by seeding human ECs (e.g., HUVEC) together with MSCs on carrier materials (85, 96). Stringent quantification of both human MSCs-derived stroma and human blood populations in humanized ossicles is difficult to be performed and required the application of most sophisticated imaging strategies. Most of the reported ossicle approaches are based on the use of primary MSCs isolated from BM of healthy donors. Specifically, these cells are very heterogeneous in terms of proliferation and differentiation capacity and this reflects not only the source- and the donor-dependent variability, but also specific differences in isolation/expansion procedures (100). Furthermore, there is a striking batch-to-batch variability in their ability to form ossicles *in vivo*. All these aspects highlight some limits in the full exploitation of these models although this does not diminish the potential of humanized ossicle approaches for studying human healthy and pathological hematopoiesis.

### Application of MSC-Derived Humanized Ossicles to Study Human Normal Hematopoiesis

The establishment of a human BM microenvironment within heterotopic ossicles was associated with enhanced long-term engraftment of human HSCs, as compared to mouse bones (87, 95, 96). The human HSC engraftment was successfully supported also by BM niches generated from cord blood stromal cells (94). Notably, human MSCs, included in the ossicle, release an increased level of cytokines, such as VEGF and IL-6, that accelerates vascularization and enhances the recruitment of human HSCs (84). Moreover, an improved myeloid development was obtained compared with lymphoid-biased human engraftment usually observed in the BM of intravenously transplanted NSG mice (85, 87, 96). Furthermore, some studies have demonstrated that humanized ossicles maintain the quiescence and the self-renewal potential of human HSCs, which can successfully engraft secondary recipient mice, with a higher efficiency compared to murine BM (87, 92). Within the ossicle, human HSCs CD45^+^CD34^+^CD90^+^ have been found in close proximity to human MSCs, suggesting that direct cell-cell contact is fundamental to regulate their fate (6). The ossicle system allows genetic manipulation of human niche components to better understand directly *in vivo* the role of specific factors critical for human hematopoietic reconstitution. Overexpression of CXCL12 by human MSCs in the ossicle results in a specific enrichment in common myeloid progenitors, megakaryocyte/erythrocyte progenitors, multipotent progenitors, and HSC populations expressing the CXCR4 receptor (6).

### Application of MSC-Derived Humanized Ossicles to Model Human Malignant Hematopoiesis

Patient-derived xenograft (PDX) mouse models are currently the gold standard for studying the development of human leukemia. However, engraftment and expansion of human acute myeloid leukemia (AML) *in vivo* remain challenging as a substantial number of samples fail to engraft also the most optimized host mice, particularly in the case of malignancies less aggressive (101). The reason may be that some subtypes of AML have low progenitor cell frequency or some samples may be particularly sensitive to the lack of a specific cell type in the mouse BM or a factor that is poorly or not at all cross-reactive between mice and humans. Hence, MSC-derived humanized ossicle models have raised great interest in the leukemia field, as recently extensively discussed in the review of Abarrategi et al. (83). The first study of AML engraftment in humanized microenvironment was reported by Vaiselbuh et al., who demonstrated that primary AML cells injected directly in pre-implanted scaffolds coated with human MSCs or intravenously in mice after implant, successfully engrafted in the ectopic niche (80). Further studies demonstrated that AML samples non-engrafting in mouse BM, such as acute promyelocytic leukemia (APL), were able to efficiently engraft in the humanized microenvironment (87, 96). Importantly, ossicles maintained the clonal heterogeneity in xenografted AML cells and their stem cell self-renewal capacity better than murine BM, as demonstrated by serial transplantation assays (87, 89, 90). Humanized BM ossicles are useful also for the engraftment of small myeloid clones, such as TP53 mutated AML subclones (102). Battula et al. developed a different approach called “human bone implant” that consists of subcutaneous transplants in NSG mice of fragments from freshly collected human BM biopsies using Matrigel® as a carrier (103). The implanted human BM tissue undergoes vascularization and bone restoration in mice, providing a functional human BM microenvironment capable of supporting the human AML engraftment. In addition to AML, a humanized ossicle system has been used to engraft primary multiple myeloma patient samples, which are known to be highly dependent on the human BM microenvironment for their survival and growth (88). Furthermore, humanized ossicles facilitated robust engraftment of myelofibrosis specimens, which has previously shown only limited engraftment with transplantation of large numbers of patient-derived CD34^+^ cells in conventional xenograft models (87). Genetical manipulation of human niche components can likely help in better understanding the role of factors critical for leukemia engraftment/progression. Deletion of hypoxia-inducible factor (HIF)-1α in human MSCs impaired leukemia engraftment in BM organoids by decreasing CXCL12 expression (85). Another pilot study demonstrated that the blockade of NF-kB activation through IkBα-SR overexpression in MSCs in the humanized ossicle model reduces leukemia burden following chemotherapy, diminishing the stroma-mediated chemoresistance (86). Carretta et al. improved the development of the myeloid compartment from leukemic samples by genetically engineering human MSCs to express IL-3 and TPO (67). Recently, a fully humanized hematopoietic niche system has been exploited to investigate the multidirectional crosstalk among AML, HSCs and the microenvironment and allowed to identify stanniocalcin 1 and its transcriptional regulator HIF-1α as specific mediators whereby AML impairs normal hematopoiesis by remodeling the mesenchymal niche (104). Of note, current 3D models use MSCs isolated from BM of healthy donors, which are molecularly and functionally different from disease-exposed ones. The use of patient-derived niche components may further improve these models and help unravel the role of the niche in the development of hematopoietic diseases. We recently reported an AML stromal niche model obtained using MSCs derived from BM of AML patients (105). AML-MSCs derived ossicles contained a significantly increased fraction occupied by adipocyte and represent an osteoprogenitor-rich niche with the presence of osterix^+^/osteocalcin^−^ pre-osteoblasts and osteocalcin^+^/Dentin matrix acid phosphoprotein (DMP) 1^−^ immature osteocytes that correlated with the reduced mature bone formation. However, the generation of humanized ossicles from MSCs and hematopoietic cells from the same patient in an autologous setting has yet to be demonstrated but it would provide a personalized *in vivo* model to test new therapies.

## Recapitulating T Cell Ontogeny Using MSCs

T cells originate from BM derived lymphoid progenitors differentiating in the thymus. The 3D organization of the thymus is provided by different cell types and creates a complex unique environment for T cell development (106). There are numerous reasons and motivations to recapitulate T cell education in the context of thymic organoids.

- Primary immunodeficiencies constitute a major cause of deficiencies. BM or umbilical cord transplant represent a clinical approach that is potentially limited by the onset of graft vs. host disease (GvHD) and slow reconstitution of the T cell compartment (107). Also, patients who underwent thymectomy or suffer from the DiGeorge syndrome, a genetic disorder underpinning thymus hypoplasia resulting from microdeletion on chromosome 22, would benefit from thymus bioengineering.
- Thymic involution. After reaching the maximum size during adolescence, the thymus begins to shrink and T cell generation decreases in a process called “thymus involution” (108). Thymic involution is associated with aging and exacerbated by several pathological and environmental influences including viral and bacterial infections, drugs or irradiation, affecting its functionality and leading to a decline in naïve T cell output (109, 110). Defective thymus structure and dysfunction negatively influences the adaptive immune system (110). Therefore, regenerating thymic function through replacing a defective thymus by an artificial thymus organoid is of high clinical interest for overcoming potential immunodeficiency or malignancies and maintaining the adaptive immune system.
- Adoptive T cell therapy using infusion of antigen-specific T cells is a promising approach in personalized medicine for the treatment of cancer or chronic viral infections. Engineering TCR-specific T cells starting from CD34^+^ HSPCs (111–113) or T cell precursors derived from induced pluripotent stem cells (iPSCs) (49, 114–116) represent a promising approach that necessitates to recapitulate T cell ontogeny.
- The engraftment of thymic organoids into humanized mice is a promising approach for the induction of T cell tolerance against transplanted tissue (110).
- Mechanistic studies. *In vitro* models of thymic education offer unique advantage to study and mechanistically dissect thymic selection. For instance, *in vitro* systems are particularly amenable to live imaging approaches (117).

T cells differentiate from BM derived Lin^−^CD34^+/int^CD38^−^CD45RA^+^ progenitors seeding the thymus (118, 119), within the thymus T cell progenitors upregulate CD7, CD1a, and CD4 to generate immature single positive (ISP) cells. ISP cells further develop to CD4^+^CD8^+^ double positive (DP) cells that ultimately differentiate into CD8^−^CD4^+^ or CD8^+^CD4^−^ single positive mature T cells (SP). Thymic epithelial cells mediate positive and negative selection of T cell progenitors cells (TEC). TEC can be classified in cortical (cTEC) and medullary (mTEC) epithelial cells. cTEC deliver chemotactic (e.g., CCL25, CXCL12), differentiation (e.g., DLL4), and survival (e.g., IL-7, SCF) signals to developing T-cells undergoing positive selection (120, 121). mTEC express AIRE, together with dendritic cells, present self-antigens ensuring the deletion of high affinity self-reactive T-cells (122).

**Figure 3:**
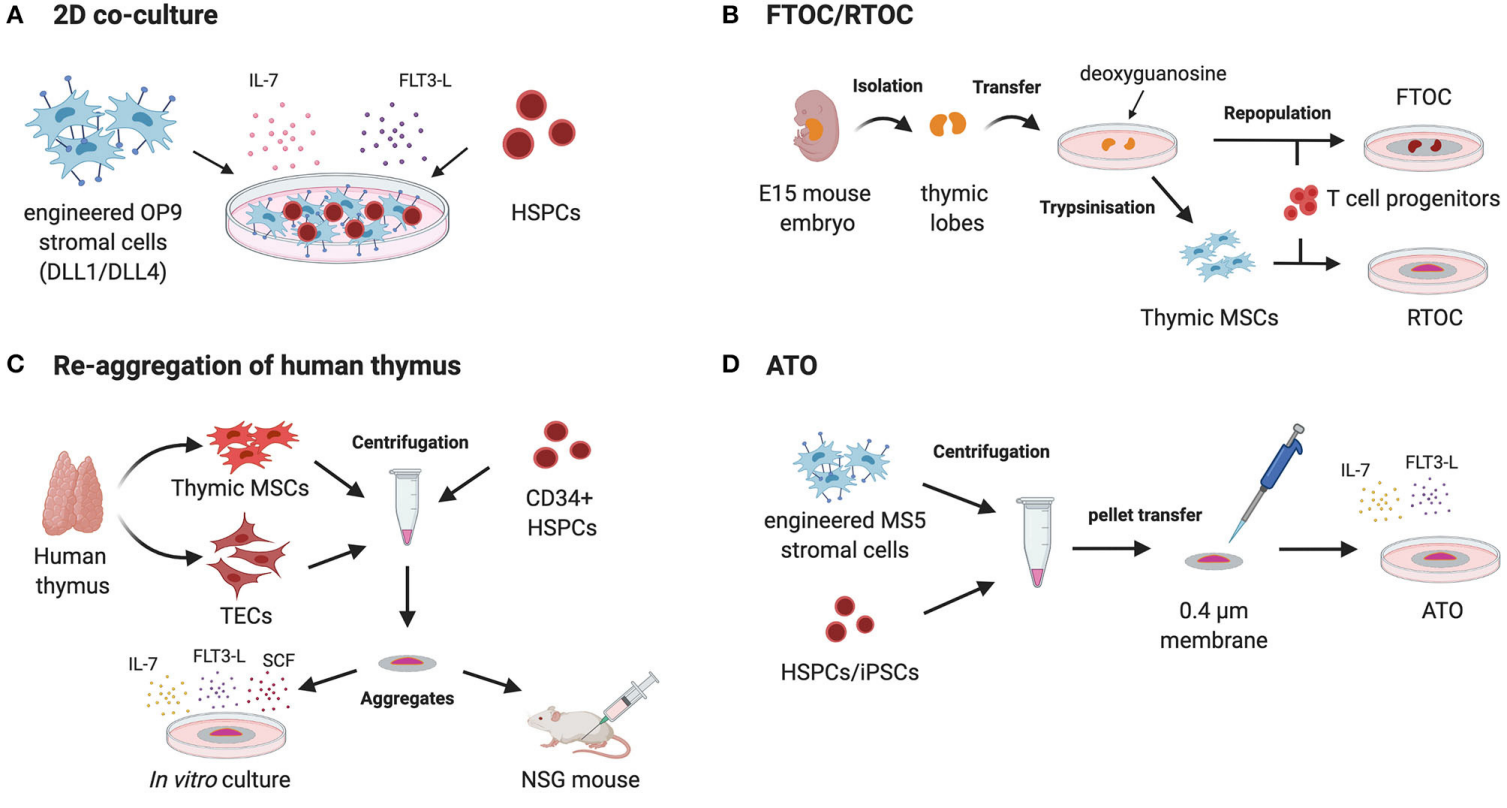
Different strategies to recapitulate T cell ontogeny using MSCs. 2D co-culture of HPCs (hematopoietic progenitor cells) on bioengineered MSC-derived OP9 cells expressing Notch ligands DLL1/DLL4 with recombinant IL-7 and FLT3-L **(A)**. Isolation of murine thymic lobes from day 14 to 15 old mouse embryos followed by their *in vitro* culture with deoxyguanosine for 5–7 days to deplete intra-thymic T cells. The thymic lobes are then repopulated by different T cell progenitors and cultured on the surface of membranes as fetal thymic organ culture (FTOC). For reaggregation thymic organ culture (RTOC) thymic stromal cells (thymic SCs) are extracted from the lobes and reaggregated with T cell progenitors by centrifugation. The cell suspension is cultured on a filter membrane *in vitro* **(B)**. Post-natal human thymus is dissociated in TECs and thymus mesoderm (TM) and further reaggregated with human CD34^+^ cells leading to the formation of thymic organoids. These organoids support the development of mature human T cells when kept in culture or when engrafted within the quadriceps muscle sheath of NSG mice **(C)**. Artificial thymic organoids (ATOs) are generated by the centrifugation of bioengineered MS5 cells expressing Notch ligands DLL1/DLL4 with HSPCs or iPSCs, respectively. After resuspension in a small amount of culture medium the cell suspension is placed on a membrane at the air-liquid interface to form 3D aggregates. This method recapitulates human lymphopoiesis and offers the ability to generate conventionally naïve T cells from HSPCs or iPSCs *in vitro* **(D)**.

Most experimental systems aiming at modeling thymus function relies on the manipulation of TECs and fall outside of the scope of this review focusing on mesodermal components of hematopoietic niches (123). In brief, 2D culture of TECs provided disappointing results in generating lymphocyte progenitors in line with the loss of primary phenotype upon *in vitro* culture (124–126) (Table 4). First attempts of 3D cultures were based on murine fetal thymic organ culture (FTOC) (140, 141) (Figure 3B). Despite the successes in supporting T cell development, FTOC evolved into reaggregate thymus organ culture (RTOC) (141). RTOC allows to manipulate the cellular composition and thereby to study the role of specific cell types, pathways or key signals including the Notch Delta like ligand (123) (Figure 3B).

**Table 4:** Stromal systems recapitulating T cell maturation.

**Reference**	**Approach**	**Findings**
Röpke (124), Masuda et al. (125), and Corbeaux et al. (126)	Culture of TECs on feeder cells	Notch signaling pathway from thymocytes to TECs is involved in TECs maturation and lymphoid development.
Schmitt and Zuniga-Pflucker (127)	Co-culture of OP9 expressing hDLL1 with fetal liver progenitors with addition of IL-7	Differentiation toward formation of α/β and γ/δ T cells
La Motte-Mohs et al. (128)	CD34^+^CD38^−^ HSPCs cultured on OP9-DL1	Appearance of CD7^+^ pro-T cells, CD4^+^ intermediate SP, and CD4^+^CD8^+^ DP
Yeoman et al. (129)	Murine FTOC seeded with human CD34^+^ HSPCs (human UCB or BM HSPCs)	Formation of human T cells which can rapidly develop into CD4^+^ or CD8^+^ SP cells expressing CD3
Poznansky et al. (130), Traggiai et al. (131), Ishikawa et al. (132), and Pearson et al. (133)	Tantalum-coated carbon matrix embedded with murine thymic epithelial cells and human cord blood CD34^+^ HSPCs	This system supports the differentiation of SP CD4^+^ or CD8^+^ mature T cells able to respond to mitogens
Chung et al. (134)	Dissociation and re-aggregation of post-natal human thymus in TEC and thymus mesoderm forming thymic organoids seeded with CD34^+^ HSPCs	When engrafted within the quadriceps muscle sheath of NSG mice thymic organoids are seeded by T cell precursors (from the cord blood origin). Furthermore, the organoids support differentiation of T cells exhibiting a broad repertoire of TCRβ chains
Parent et al. (135)	Development of human thymic epithelium from iPSCs has opened new avenues for the production of thymic organoids	Human ESCs-derived thymic epithelium supports the development of murine T cells within thymus-deficient mice
Melkus et al. (136), Wege et al. (137), and Kalscheuer et al. (138)	Implantation of fetal thymus under the renal capsule leads to the formation of a competent thymus subsequently seeded by BM HSPCs delivered intravenously	The organoid supports the full maturation of T cells in 15-20 weeks
Seet et al. (139) and Montel-Hagen et al. (116)	3D artificial thymic organoids (ATOs) composed by ectopically expressing DLL4 murine BM MSC line MS5 and HSPCs or iPSCs	This method recapitulates human lymphopoiesis. T cells display a normally broad repertoire and exhibit normal responsiveness (proliferation, cytokines) upon TCR triggering

*TEC, thymic epithelial cells; HSPCs, hematopoietic progenitor stem cells; SP, single positive; NSG; TCR, T cell receptor; MSC, mesenchymal stem cells; iPSCs, induced pluripotent stem cells; UCB, umbilical cord blood; ATO, artificial thymic organoid; TCR, T cell receptor. BM, bone marrow; DLL1/4, Delta Like Notch Ligand 1/4; FTOC, fetal thymic organ culture; DP, double positive*.

Mesenchymal stromal cells are part of the physiological thymic microenvironment. Recent single cell studies have highlighted the complexity of the mesenchymal compartment of the thymus. Thymic fibroblasts can be distinguished in type 1 (Fb1) and type 2 (Fb2) fibroblasts (142). Fb1 cells are characterized by the expression of an important key player in innate immunity termed COLEC11 as well as by the expression of the enzyme ALDH1A2 that controls the production of retinoic acid functioning as an epithelial growth regulator. On the other hand, Fb2 cells are characterized by ECM genes as well as semaphorins that regulate vascular development. For that reason Fb2 cells are mainly found close to large blood vessels lined with VSMCs (142). ECM produced by thymic MSCs might play a crucial role in the maintenance of TEC phenotypes. For instance, human fibroblast/MSCs uniquely provides an environment supporting promiscuous gene expression by mTECs associated to AIRE and FoxN1 expression (143).

Here we provide examples of the implementation of MSCs in modeling T-cell education and thymic function.

### Harnessing MSCs to Recapitulate T Cell Education

The Zúñiga-Pflücker lab has pioneered the implementation of murine MSCs engineered to express NOTCH ligands to drive T-cell maturation from HSPCs. OP9 is MSC line derived from CSF1-deficient mice with a broad hematogenic activity dependent on SCF/KITL expression (144) but unable to sustain the generation of T cells (127). Schmitt et al. have shown that ectopic expression of the Delta-Like-1 (DLL1) NOTCH ligand is sufficient to confer the ability to support ontogeny of α/β and γ/δ T cells from fetal liver progenitors in the presence of FLT3L and IL-7 growth factors (127) (Figure 3A). DLL1 provides a key signal for T cell specification at the expense of B cell development. This approach was later found to also recapitulate the ontogeny of human T cells from umbilical cord blood (UCB) CD34^+^ HSPCs via the ordered appearance of CD7^+^ pro-T cells, CD4^+^ intermediate SP, and CD4^+^CD8^+^ DP (128). Importantly, the OP9 approach (using DLL4) could also be implemented to generate T cells from human embryonic stem cells (145). However, one major drawback of the OP9-DLL system is that T cell differentiation and maturation of TCR^+^ SP CD4^+^ or CD8^+^ T cells remained inefficient for most sources of human pre or post-natal HSPCs with the exception of UCB (146). Similar to the *in vitro* TECs culture, the culture of Notch ligand expressing OP9 cell lines shows the drawback of lacking a 3D architecture (147). The BLT (BM, liver, thymus) model of humanized mice provides an option to model human thymus function *in vivo*. Implantation of human fetal thymus under the renal capsule leads to the formation of a competent thymus that can be seeded by BM HSPCs delivered intravenously to support the full maturation of T cells in 15–20 weeks (136–138) (Table 4).

Bypassing the need for fetal thymus mesodermal components have been shown to help building 3D models of human thymus. Using the dissociation and re-aggregation of post-natal humans TECs and thymus mesoderm Chung et al. have recapitulated thymus function (134) (Figure 3C). Those thymic organoids supported the development of mature human T cells when seeded with human CD34^+^ HSPCs (134). This system offers the advantage to be amenable to lentiviral transduction for manipulation of the thymic environment and bypass the use of xenogenic (murine) thymic epithelium (134). When engrafted within the quadriceps muscle sheath of NSG mice previously reconstituted with human CB-CD34^+^ HSPCs, thymic organoids are seeded by T cell precursors (from the CB origin). Furthermore, the organoids support differentiation of T cells exhibiting a broad repertoire of TCRβ chains (134).

More recently, Seet et al. have shown that MSCs can help modeling thymus function (139). Seet et al. report a method for the formation of artificial thymic organoids (ATOs) in which TECs were replaced by the ectopically expressing DLL4 murine MSC line MS5 (MS5_DLL4) and centrifuged with HSPCs or iPSCs, respectively (116, 139) (Figure 3D). ATO simulate the 3D structure of the thymus and can be compared to RTOC (139). After resuspension in a small amount of culture medium the cell suspension was dropwise placed on a membrane at the air-liquid interface to form 3D aggregates. This method recapitulates human lymphopoiesis and offers the ability to generate conventionally naïve T cells from ESCs or iPSCs, respectively (116, 139). Furthermore, this system enables long-term culture and provides improved positive selection due to the 3D organization. T cells developing in ATOs display a normally broad repertoire and exhibit normal responsiveness (proliferation, cytokines) upon TCR triggering. Of crucial relevance for clinical application, this method is amenable to generate TCR-transduced T cells generated after efficient allelic exclusion at the Vβ locus (116, 139). This has been exemplified using TCR specific for NY-ESO or MART1 tumor-associated antigens (116, 139). In sum, the ATO system demonstrates the high versatility of engineered MSCs to recapitulate cellular interactions underlying T cell development.

## Conclusion and Future Directions

This review highlights the potentially vast range of application for MSCs in the engineering of immune niches supporting leukocyte development. Among the multiple technological challenges raised by the implementation of MSCs, some salient topics emerge defining possible future directions in the field.

### Understanding MSCs Developmental Phenotypic and Functional Heterogeneity to Build Better Niches

Recent developments in high dimensional approaches, such as unbiased scRNAseq have brought a fresh look on the heterogeneity of MSCs associated to immune niches (19–22, 148–151). For instance, whole genome expression analysis reveals an exquisite specificity in the distribution of niche factors among the diverse MSC types (22). Important development in spatial transcriptomics and live imaging should unravel the spatial organization of intracellular interactions supporting the function of MSCs within niches (21, 152). Deciphering the functional impact of MSCs heterogeneity and the division of labor between different MSC types should substantially assist tissue engineering purposes.

### Engineering MSCs to Improve Their Function

MSCs can be genetically manipulated *ex-vivo* to modulate the expression of key molecules before they are embedded within immune niches. This approach has been developed with success for the establishment of immune niches supporting AML engraftment (67) or supporting the development of dendritic cells (68) in humanized mice. Further programming of MSC transcriptional landscapes (63) broadly impacting on their function might open new avenues for the engineering of immune niches.

### Harnessing the Differentiation Pathway of Endogenous Progenitors for MSCs

Understanding the developmental pathways of MSCs populations is of major relevance for tissue engineering. The phenotype and function of differentiated MSC types is potentially difficult to maintain in *ex vivo* cultures (63). Therefore, approaches co-opting the physiological developmental pathway of MSCs are of particular interest. For instance, disentangling the developing hierarchies within early MSC progenitors underpinning the BM niche (30–32, 153) should facilitate the technological implementation of MSCs to build synthetic niches and organoids.

### Stimuli-Responsive Dynamic Immune Niches

An essential biological feature of immune niche is their ability to respond dynamically to immune perturbations. BM, for instance, also respond to acute inflammation, often by increasing myelopoiesis, a process termed as “emergency myelopoiesis” (154). In both cases dynamic changes of hematopoietic organs rely on adaptation of the stromal network (19, 22, 150, 151, 155, 156). Assessing this responsiveness feature should be of interest to recapitulate leukocyte development associated to inflammatory settings.

### Patient-Specific Immune Niches for Drug Testing

Genetic variation is likely to impact on physiological niches function. One key feature of the BM niche ossicle model is the possibility to transfer in the mouse the human BM microenvironment, either normal or pathological. For instance, the ossicle models represent a valuable tool to unravel the role of cellular and molecular mechanisms underlying the interactions between the hemopoietic and stromal compartment in normal or pathological niche. Ultimately, this analysis could be performed using MSCs and malignant cells from the same patient thereby defining a platform for drug screening. This approach could be applied to targeted therapies interfering with stromal support.

## Author Contributions

RS, AP, EM, and JP contributed equally to the manuscript writing and figure generation. AG, GA, JU, and PB helped during the manuscript writing and revision. MS and PG supervised the work and took part in the writing process. All authors contributed to the article and approved the submitted version. All figures were created by RS with BioRender.com.

## Conflict of Interest

The authors declare that the research was conducted in the absence of any commercial or financial relationships that could be construed as a potential conflict of interest.
